# The Role of Postural Assessment, Therapeutic Exercise and Foot Orthoses in Haemophilic Arthropathy: A Pilot Study

**DOI:** 10.3390/life15081217

**Published:** 2025-08-01

**Authors:** Dalila Scaturro, Sofia Tomasello, Vincenzo Caruso, Isabella Picone, Antonio Ammendolia, Alessandro de Sire, Giulia Letizia Mauro

**Affiliations:** 1 Precision Medicine in the Medical, Surgical and Critical Care Areas, University of Palermo, 90100 Palermo, Italy; dalila.scaturro@unipa.it (D.S.);; 2 Villa Delle Ginestre, Palermo Provincial Health Authority, 90146 Palermo, Italy; 3 Section of Pharmacology, Department of Biomedical and Biotechnological Sciences, University of Catania, 95124 Catania, Italy; 4 Physical Medicine and Rehabilitation Unit, Department of Medical and Surgical Sciences, University of Catanzaro “Magna Graecia”, 88100 Catanzaro, Italy; 5 Research Center on Musculoskeletal Health, MusculoSkeletalHealth@UMG, University of Catanzaro “Magna Graecia”, 88100 Catanzaro, Italy

**Keywords:** haemophilia, foot orthoses, rehabilitation, hemarthroses, hematomas

## Abstract

Haemophilic arthropathy is caused by repeated joint bleeding episodes, primarily affecting knees, ankles and elbows. Conservative options should be considered prior to surgery, as well as postural evaluation, since any functional overload promotes the development of new bleeding. The aim of this study is to verify the use of foot orthoses in combination with postural rehabilitation, assessing the incidence of spontaneous haemarthroses and haematomas. In total, 15 patients were enrolled and randomly divided into two groups: 8 in group A, composed of patients who were prescribed foot orthoses and a 20-session rehabilitation program, and 7 in group B, composed of patients who were instructed to use foot orthoses only. All patients were evaluated at baseline (T0), at 3 months (T1—end of the rehabilitation program), and at 12 months (T2), using the following scales: Functional Independence Score in Haemophilia (FISH), Haemophilia Joint Health Score (HJHS) and Numerical Rating Scale (NRS). During the 12 months between the first and the last assessment, no patient in group A developed hemarthroses or hematomas, while one case of hemarthrosis was recorded in group B. The HJHS improved significantly (≤0.05) in group A at both T1 and T2, while in group B it improved significantly only in T2. As for FISH, it showed significant improvements in both groups at T1 and T2. NRS showed a significant reduction only at T2 in both groups (*p*-value T0–T1 0.3 in group A e 0.8 in group B). No patient reported any adverse effects from the use of orthotic insoles. The combination of postural rehabilitation, the use of foot orthoses and pharmacological prophylaxis could improve functioning and joint status in patients affected by haemophilic arthopathy, delaying or preventing new hemarthroses by improving the distribution of joint loads and the modification of musculoskeletal system’s characteristics.

## 1. Introduction

Haemophilia is the most common severe hereditary haemorrhagic disorder and it is caused by the deficiency or dysfunction of coagulation factors in the blood [1]. There are two main types of haemophilia: haemophilia A, most frequent, which occurs due to low amounts of clotting factor VIII, and haemophilia B, which occurs due to low levels of clotting factor IX [2].

Factor VIII and IX levels must be >40% to allow normal haemostasis. The functional level of factor VIII or IX in haemophilia A and B and therefore the severity of the bleeding, varies depending on the specific mutation in the gene of the deficient factor. According to this level, three severity degrees of haemophilia can be distinguished: mild (if the functional activity of the deficient factor is between 5 and 40% of normal), moderate (between 1 and 5%) and severe (<1%) [3]. Therapeutic management primarily involves replacing the deficient coagulation factor to restore adequate haemostasis. In cases where signs and symptoms suggest active bleeding, prompt initiation of treatment is essential. In addition to plasma-derived and recombinant factor concentrates, certain adjunctive agents can play a supportive role in specific scenarios. Antifibrinolytic drugs—such as tranexamic acid and epsilon aminocaproic acid—are particularly indicated in managing mucosal bleeds (e.g., oral, nasal, gastrointestinal, or genitourinary), where fibrinolysis tends to be more pronounced. These agents help stabilize formed clots and are often used as an adjuvant to factor replacement. Desmopressin (DDAVP) is also useful in selected patients, especially those with mild haemophilia A, as it can transiently increase endogenous factor VIII levels [4,5].

Haemophilic arthropathy (HA) represents the most common long-term complication in individuals with haemophilia and is frequently linked to a significant decline in quality of life. The condition arises as a result of recurrent bleeding into the joints, which triggers chronic proliferative synovitis and gradually damages the articular cartilage. The joints most involved are the knees, ankles and elbows. Lower limb arthropathy leads to walking deficits, marked muscle hypotrophy, ex non usu, with hyposthenia and instability, which lead to severe impairment of autonomy and quality of life (QoL). For this reason, HA is the main cause of morbidity in haemophilic patients [6].

From a pathophysiological point of view, there are two mechanisms that lead to cartilage destruction: a direct process, represented by the altered metabolism of cartilage as a consequence of intra-articular bleeding, and an indirect mechanism, whereby the accumulation of haemosiderin in synovial macrophages causes synovial hypertrophy, which is followed by lysosomal enzymes’ release from synovial cells and thus cartilage destruction [7].

The diagnosis of haemophilic arthropathy is based on clinical evaluations and imaging findings. The objective examination assesses the presence of swelling, crepitus, instability and range of motion. However, the main role is played by instrumental investigations [8]. Ultrasound (US) is a key tool for diagnosing and monitoring haemophilic arthropathy (HA), offering early, non-invasive and cost-effective detection of joint damage resulting from recurrent bleeding. The use of point-of-care ultrasound (POCUS) further enhances clinical utility by allowing real-time bedside evaluation, especially in acute settings. Additionally, the standardized HEAD-US protocol improves consistency and reliability in assessing commonly affected joints—elbows, knees and ankles—with findings that correlate well with MRI [9]. X-rays are typically the first imaging technique used to assess haemophilic arthropathy due to their ability to provide a comprehensive view of the joint at a relatively low cost. They are particularly effective in identifying moderate to severe arthropathic features, including joint space narrowing, irregularities in the subchondral bone and subchondral cyst formation. X-rays are also recommended every 5 years for monitoring the overall condition of the joint. Magnetic resonance imaging, on the other hand, is the most sensitive imaging modality for evaluating soft tissue and cartilage changes and is therefore considered the gold standard for HA evaluation [10].

A key role in the management of these patients is played by prophylaxis. Many patients with severe haemophilia receive regular infusions of factor VIII or factor IX to prevent bleeding episodes. The goals of modern haemophilia management are to minimise joint disease and maximise quality of life. In many cases, this can be achieved by starting prophylaxis early. Most standard prophylaxis regimens are designed to maintain minimum factor levels > 1%.

Management of acute haemarthrosis involves prompt replacement of the deficient coagulation factor, adequate pain control and rest. The objective of factor replacement therapy is to achieve haemostatic levels (30–50% factor activity) sufficient to halt bleeding. Treatment recommendations for patients who develop chronic synovitis and arthropathy must be made after careful evaluation of all potential options. Conservative options to minimize bleeding and pain control should be considered prior to surgery [11,12].

Surgery should be performed only after establishing the feasibility and availability of both prolonged postoperative factor replacement and rehabilitation. Depending on this, several rehabilitative approaches can be considered for the management of these subjects, including analgesic therapy, physiotherapy, synovectomy, steroid and hyaluronic acid injections, joint arthroplasty and arthrodesis [13,14,15].

Another element to be considered in the therapeutic setting of these patients is the posture, since any functional overload on the joints most affected by the disease is an important factor favouring the development of new bleeding. For this reason, the use of foot orthoses is very useful since they allow for better redistribution of loads, thus avoiding excessive joint stress and promoting greater stability of the joints, as shown in a study by De la Corte-Rodriguez et al., where the use of foot orthoses in the management of HA patients was evaluated [16].

The aim of our pilot study is to verify the efficacy of combining postural rehabilitation, consisting of 20 sessions of exercises based on the Back School method, and use of foot orthoses, consisting of custom-made proprioceptive insoles in polyethylene with lateral edges, longitudinal vault support and medial or lateral wadge. The primary endpoint is the prevention of haemarthroses (intra-articular bleeding) and haematomas (extra-articular bleeding), while the secondary endpoints consist of enhanced functional independence in patients with haemophilia as well as pain reduction and improved function of the joints most commonly affected by haemophilic arthropathy, namely ankles and knees.

## 2. Materials and Methods

### 2.1. Test Design

We conducted a pilot study on outpatients affected by haemophilic arthropathy attending the UOC of Recovery and Functional Rehabilitation of the AOUP Paolo Giaccone of Palermo, Italy, sent by the UO of Haematology of the same University Hospital, in a period from January 2017 to March 2023.

**Figure 1:**
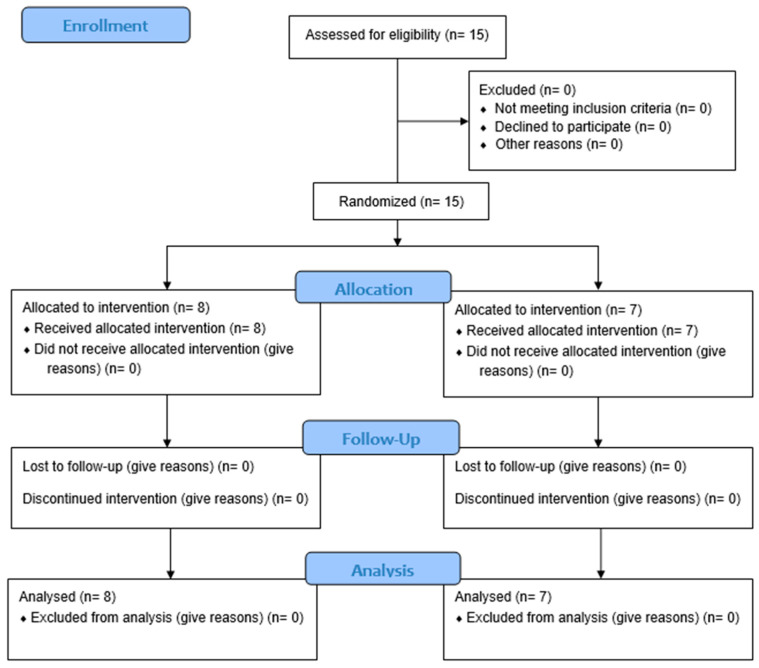
CONSORT flow diagram.

The study received the approval of the local ethics committee ‘Palermo 1’ (approval no. 04/2023) of the AOUP Paolo Giaccone in Palermo and was conducted in accordance with the Declaration of Helsinki and the CONSORT Guidelines (Figure 1). Information and data were processed according to Good Clinical Practice (GCP) guidelines.

All patients enrolled in the study received and signed an informed consent form for participation.

Ethics committee approval date: 19 April 2023; Clinical trial registration number: NCT06438406; Clinical trial registration date: 27 May 2024.

### 2.2. Participants

The inclusion criteria used were the following: diagnosis of severe haemophilia A or B; age ≥ 6 years and ≤25 years; presence of hindfoot and/or arch misalignment; prophylaxis with factor deficient (VIII or IX). Patients were excluded if they had prosthetic implants/synthetis means or if they were uncooperative.

The presence of hindfoot and/or arch misalignment was evaluated through a careful clinical examination including posterior observation in the upright position, Heel Rise Test and evaluation with a polarized light LED podoscope. Additionally, all patients underwent a baropodometric examination aimed at determining the most appropriate foot orthoses prescription.

### 2.3. Intervention

The study population was randomly allocated into two distinct groups using a computer-generated randomization sequence in order to minimise allocation bias and ensure comparable baseline characteristics. Group A (Gr. A) included patients who received a dual intervention consisting of custom-made foot orthoses and a structured rehabilitation program comprising 20 individual sessions. This program aimed to improve postural alignment and joint function. Group B (Gr. B), on the other hand, included patients who were prescribed only foot orthoses, without the addition of any rehabilitative exercise protocol. This grouping allowed for the evaluation of the potential added benefit of postural rehabilitation when combined with orthotic treatment.

Foot orthoses were prescribed following a baropodometric assessment to select the most appropriate device for each patient’s specific condition. Prior to use, the orthoses were checked by the research team to ensure proper fitting and adherence to the prescribed specifications and intended therapeutic purpose.

Patients were instructed to use their orthoses gradually and to continue with their usual lifestyle.

All patients were prescribed custom-made proprioceptive insoles in polyethylene with lateral edges and longitudinal vault support and in addition, depending on the characteristics found on the baropodometric examination, a medial wedge in patients with valgism or a lateral wedge in patients with varism, with the aim of promoting realignment of the ankle axis.

### 2.4. Rehabilitation Program

The rehabilitation program was based on the Back School method, which aims not only to reduce pain but, more importantly, to address its underlying causes. This program was carried out individually on patients with respect to the emerging problems assessed by clinical examination, with the following objectives: prevent haemarthrosis/haematoma, recover/maintain range of motion, recover/maintain muscle strength, prevent joint deformities, improve posture and walking. The rehabilitation protocol consisted of 20 standardized sessions, each lasting 60 min and delivered twice weekly. The intervention was based on the Back School method, integrating analgesic strategies with functional postural re-education. The therapeutic program included:

- postural retraining exercises aimed at automating correct static and dynamic alignment;
- core stabilization techniques and neuromuscular control exercises designed to enhance spinal protection under mechanical stress;
- compensatory exercises to restore spinal balance in the sagittal and frontal planes, particularly in patients exposed to prolonged occupational or sports-related postures or stresses;
- targeted muscle mobilization and myofascial stretching protocols to address segmental stiffness and muscular imbalances;
- specific exercises addressing hindfoot malalignment, aimed at improving joint stability and proprioception through active mobilizations and targeted strengthening of the posterior tibialis, peroneal muscles and plantar compartment.

In accordance with this approach, the study by Cuesta-Barriuso et al. demonstrated that a self-administered myofascial release protocol may produce improvements in pain intensity, range of ankle motion in plantar flexion and overall functional capacity in individuals with haemophilia [17].

This program was also combined with proprioceptive training using wobble boards, uneven surfaces and visual biofeedback, which, according to Wagner et al., helps prevent relapses [18].

All haemophilic patients continued prophylaxis according to the protocol prescribed by the haematologist. The use of COX-2 inhibitors and/or analgesics was not allowed throughout the physiotherapy period in order to avoid any confounding effects on the study results.

### 2.5. Outcome Measures

All patients were assessed at baseline (T0), at 3 months (T1—at the end of rehabilitation treatment) and after 12 months (T2).

These assessments were always performed by the same physiatrist, with 15 years of experience in the rehabilitation setting of patients with HA, who was unaware of the group to which the patient visited belonged.

Main outcome measures were specific arthropathic scales, namely the Haemophilia Joint Health Score (HJHS), reflecting function and status of the knee, ankle and elbow, and the Functional Independence Score in Haemophilia (FISH), related to the functional ability of patients, were used to assess outcomes. Moreover, we also used the Numerical Rating Scale (NRS) to quantify the knee and ankle pain intensity.

In conclusion, the postural evaluation was conducted with the patient in a static position, analysing their alignment across various planes and taking advantage of the APECS (AI Posture Evaluation and Correction System^®^) mobile app [19].

During re-evaluations, any appearance of new haemarthroses and haematomas was also assessed through knees and ankles ultrasound examination.

The Functional Independence Score in Haemophilia is a performance-based assessment tool used to measure functional independence in daily activities. It consists of 8 domains (eating, hygiene, dressing, sitting, stooping, walking, stairs, running) divided into 3 groups, each of which is assigned a score ranging from 1 to 4, depending on the individual’s greater or lesser ability to perform that specific function. The maximum score is 32 (the highest level of autonomy) [20].

The Haemophilia Joint Health Score is a rating scale that measures joint health in the domains of anatomical structure and function (biomechanics) of the joints most frequently affected by bleeding in haemophilia: knees, ankles, elbows. The presence of oedema, atrophy, crepitus, contractures in flexion, axial deformities, loss of mobility and instability are searched for; points are assigned to each relief according to severity and added together. It gives a maximum score of 124 points (a higher score is worse) [21,22].

The NRS scale is a one-dimensional 11-point scale that assesses pain intensity in adults, with a range from 0 to 10, corresponding to ‘no pain’ and ‘worst pain imaginable’. The patient indicates the intensity of his or her pain verbally. Other versions exist in the literature, but the 11-point scale remains the most widely used. The limitation of the scale is that, being one-dimensional, it is more suitable for assessing current pain or pain experienced in the last 24 h, being less appropriate when symptoms fluctuate [23,24].

APECS (AI Posture Evaluation and Correction System^®^) is a mobile app that, by analysing photos of the subject under examination in its various projections—anterior, posterior and lateral—allows a detailed postural evaluation thanks to the use of precise photogrammetric algorithms. Using APECS, it is also possible to perform a dynamic postural assessment, by means of video analysis of the posture in lateral view and evaluation of angles and movements [19].

### 2.6. Statistical Analysis

All data collected in the study were summarized as mean ± standard deviation. In both groups (A and B), analysis of the HJHS, FISH and NRS parameters was performed using the Wilcoxon signed-rank test for paired data, with Bonferroni correction applied for multiple comparisons. This approach was chosen due to the limited number of patients in both groups. Given the small sample size and in order to enhance the robustness and interpretability of the results obtained, the effect size was also calculated for each comparison. Specifically, we used r (effect size), calculated as Z/√N, which is an appropriate and reliable measure in the context of non-parametric tests and pilot studies [25]. Statistical analyses were carried out with the support of a statistical expert from our institute, using SPSS software version 15.0 (SPSS Inc., Chicago, IL, USA). Given the rarity of the disease and the related limitations in recruitment, the study was designed as a pilot study, with a statistical power of 17% to detect an absolute difference of 15%. Therefore, results should be interpreted with caution, pending larger studies.

## 3. Results

From the data analysis, as shown in Table 1, 15 patients were enrolled and randomly divided into two groups: 8 in group A and 7 in group B. For group A, the average age at the time of the first assessment was 17.3 ± 1.7 years (range 6–25), while for group B it was 16.8 ± 3.2 years (range 8–22). The diagnoses were as follows: 12 patients had severe haemophilia A and 3 had severe haemophilia B. All patients had hindfoot misalignment, and in particular: 11 patients had rearfoot valgus and 4 had rearfoot varus. Of the 11 patients with rearfoot valgus, 4 also had flatfoot, 3 had cavus foot and the remaining 4 had a physiological footprint. Of the 4 patients with varus hindfoot, 3 presented also cavism, while 1 had a physiological footprint. Regarding sports, 6 patients engaged in regular physical activity, while the remaining 9 did not perform any physical activity. Finally, we investigated professional activity, with 13 patients being students, 1 patient being a worker and 1 being unemployed.

The general data of the patients are shown in Table 1.

All 15 patients, except for 1, wore foot orthoses continuously for the entire 12-month period and attended the follow-up. The results of the reassessments for the two groups in terms of presence of haematoma/haemarthrosis, joint health (HJHS), functional capacity (FISH) and pain control (NRS) during the follow-up are reported in Table 2 as mean ± standard deviation.

A more detailed summary of the statistical analysis, including the *p*-values calculated using the non-parametric Wilcoxon signed-rank test, as well as the corresponding effect sizes (r) and their qualitative interpretations, are reported in Table 3.

In both groups (A and B), analysis of the HJHS, FISH and NRS parameters was performed using the Wilcoxon signed-rank test for paired data, with Bonferroni correction applied for multiple comparisons. This approach was chosen due to the limited number of patients in both groups. Given the small sample size and in order to enhance the robustness and interpretability of the results obtained, the effect size was also calculated for each comparison. Specifically, we used r (effect size), calculated as Z/√N, which is an appropriate and reliable measure in the context of non-parametric tests and pilot studies.

Regarding the number of recurrences of haemarthrosis and haematomas, no patient in group A developed any during the 12 months between the first and the last assessment, unlike group B, where a new case of hemarthrosis was recorded at T2. The HJHS score for group A improved statistically significantly at both T1 and T2 compared to baseline, while in group B, a statistically significant improvement was observed only at T2, not at T1. As for functional ability measured by the FISH, a statistically significant improvement was observed in both group A and group B at both reassessments (T1 and T2) compared to baseline. Regarding knee and ankle pain, the NRS score measured at the first reassessment (T1) showed no statistically significant difference in either group, while the reduction was statistically significant in both groups at the final reassessment (T2). None of the patients reported any adverse events following the use of foot orthoses.

Finally, with respect to the effect size (r), the majority of the measurements showed large effects, supporting the strength and consistency of the observed outcomes. However, given the exploratory nature of this pilot study, even small to moderate effect sizes (e.g., r = 0.20–0.40) are considered meaningful. In early-phase research, the primary objective is not solely to achieve statistical significance, but rather to assess the direction and potential magnitude of the intervention’s impact. Therefore, these findings—regardless of the absolute size of r—provide valuable preliminary evidence and contribute to the rationale for conducting larger, adequately powered studies in the future [26].

## 4. Discussion

In recent decades, therapeutic exercise has gained an increasingly important role in the management of haemophilia patients, as it has been shown to be useful in reducing pain perception, the risk of musculoskeletal bleeding and in increasing range of motion (ROM) and muscle strength [27].

Nevertheless, it is essential to ensure that the exercises performed do not become excessive, as excessive loading may itself contribute to the onset of new bleeding episodes. The study by Vallejo et al. suggests that for this patient population, water-based training might be beneficial, as it eliminates potential overload induce.

This study indeed demonstrates that specifically designed water-based training sessions for haemophilic patients have a positive effect in terms of motor performance, as well as aerobic and mechanical capacity, without causing adverse effects [28].

Moreover, associating rehabilitation with the use of orthoses aims precisely at redistributing loads more properly and, consequently, avoiding excessive joint stress. Orthoses, in fact, can control or prevent excessive joint movement, can stabilize a specific joint or relieve the load or stress on it [29]. To maximise the benefits of this therapeutic modality, a thorough knowledge of anatomy and biomechanics is essential, as is knowledge of the devices available for the different joints that may be affected by haemophilic arthropathy [16].

In the study by Lobet et al., specifically targeting haemophilic patients with ankle arthropathy, it is confirmed that the use of orthopaedic shoes or orthotics produces significant pain relief and improved comfort in more than half of the patients, with minimal side effects [30].

As far as we know, ours is the first study analysing the combination of postural rehabilitation and use of foot orthoses in the prevention of new haemarthroses and haematomas, assessed through knees and ankles ultrasound examination. In this study, haemophilic patients were evaluated through clinical assessments of knee and ankle pain (NRS), joint impairment of the elbows, knees and ankles (HJHS), postural alignment (APECS) and functional ability (FISH). Based on the individual characteristics of each patient, the most suitable orthotic insole was prescribed. Our results showed improvements in the HJHS, FISH and NRS scores, thus supporting the effectiveness of foot orthoses use. However, while FISH and NRS improved significantly in both groups from the first follow-up (T1), the improvement in joint impairment (HJHS) was statistically significant at T1 only in Group A, whose patients, in addition to using orthoses, also underwent a rehabilitation program aimed at achieving proper postural alignment. In contrast, Group B achieved a statistically significant improvement in HJHS only after 12 months (T2).

Furthermore, in Group B, whose patients did not undergo postural rehabilitation, a recurrence of hemarthrosis occurred, a condition that did not happen in any patients of Group A.

In conclusion, our results demonstrate that the combination of therapeutic exercise and use of foot orthoses tailored to a specific dysmorphism improves functionality, joint mobility, reduces the risk of bleeding and, in the long term, reduces pain onset.

In agreement with our findings, the study by De la Corte-Rodriguez et al. shows how orthoses can alleviate mechanical shocks, reduce pain and limit deformities by stabilizing the joint [31].

In contrast to us, Richard A. et al. in their systematic review poses more caution about the real effectiveness of orthopaedic footwear and devices in the management of haemarthrosis and ankle haemarthropathy of patients with haemophilia. The main limitations that emerged from this systematic review are the heterogeneity of the methodologies used, limitations with the study designs, the small sample size examined and the limited follow-up of participants [29].

Regarding our study, it presents several advantages. Firstly, as previously mentioned, it is the first to investigate the combined effect of therapeutic exercise and foot orthoses in the prevention of haemarthroses and/or haematomas. Additionally, a notable strength is the extended follow-up period, with outcomes observed at the initial post-treatment assessment (T1) being further confirmed—and in some cases enhanced—at the 12-month follow-up (T2).

As for the limitation of the study, the main one is represented by the small number of enrolled patients, which is a direct consequence of haemophilia being a rare disease. Indeed, the estimated frequency of haemophilia is approximately 1 in 10,000 live births (haemophilia A occurs in about 1 in 5000 live male births, while haemophilia B is less common, affecting approximately 1 in 30,000 live male births) [32]. Therefore, larger, multicenter studies are desirable to confirm the results we obtained. Another limitation is that the majority of patients were still in their developmental stage at the time of evaluation; therefore, it would be important to more clearly specify whether the prescribed insole was intended to be corrective or supportive.

Finally, as already highlighted in the study by Kuijlaars IAR et al., the HJHS score is a tool that requires a lot of time to complete correctly, so the items should be standardized to shorten it and make it more easily accessible in routine clinical practice [33].

## 5. Conclusions

In conclusion, the present pilot study showed that the combination of postural rehabilitation and use of foot orthoses, in addition to pharmacological prophylaxis for coagulation factor deficiency, could improve the function and status of knees and ankles—joints commonly affected by haemophilic arthropathy—as well as the functional independence of these patients. Indeed, an adequate rehabilitative treatment by delaying and/or preventing the formation of new haemarthroses, due to the improved distribution of loads to the lower limbs can also modify functional and structural characteristics of the musculoskeletal system. However, as previously mentioned, larger multicenter studies are required to confirm our findings regarding the promising combination of these rehabilitative approaches for the prevention and treatment of patients affected by haemophilic arthropathy.

## Figures and Tables

**Table 1:** General patient data.

	Total	Group A	Group B
No. of patients	15	8	7
Age, mean ± SD	17.3 ± 1.7	18.1 ± 2.8	16.8 ± 3.2
Severe haemophilia A	12	6	6
Severe haemophilia B	3	2	1
Valgus hindfoot	11	7	4
- flatness	4	3	1
- cavus	3	1	2
- physiological	4	3	1
Varus hindfoot	4	2	2
- cavism	3	2	1
- physiological	1	0	1
Physical activity yes	6	4	2
Physical activity no	9	4	5
Students	13	7	6
Workers	1	0	1
Unemployed	1	1	0

No. (number); SD (standard deviation).

**Table 2:** Outcome measures (mean ± standard deviation).

	T0	T1	*p*-Value (T0–T1)	T2	*p*-Value (T0–T2)
Hematoma Gr. A	0	0		0	
Hematoma Gr. B	0	0		0	
Joint bleed Gr. A	0	0		0	
Joint bleed Gr. B	0	0		1	
HJHS Gr. A	9.5 ± 3.3	7.4 ± 2.4	≤0.05	7.9 ± 1.8	≤0.05
HJHS Gr. B	8.3 ± 2.1	8.1 ± 3.3	0.6	6.2 ± 1.2	≤0.05
FISH Gr. A	26.3 ± 1.1	28.5 ± 0.8	≤0.05	28.8 ± 2.4	≤0.05
FISH Gr. B	24.8 ± 1.8	27.2 ± 1.1	≤0.05	27.6 ± 2.5	≤0.05
NRS Gr. A	3.6 ± 0.2	3.2 ± 0.7	0.3	2.1 ± 0.1	≤0.05
NRS Gr. B	3.9 ± 1.8	3.7 ± 0.4	0.8	2.7 ± 0.9	≤0.05

Gr. (Group); HJHS (Haemophilia Joint Health Score); FISH (Functional Independence Status in Haemophilia); NRS (Numerical Rating Score).

**Table 3:** Wilcoxon signed-rank test results and effect sizes (r) for T0–T1 and T0–T2 comparisons.

Variable	Comparison	Z-Value	*p*-Value	N (Paired)	Effect Size (r)	Interpretation
HJHS Gr. A	T0–T1	−2.2901	0.02	8	0.81	Large
	T0–T2	−0.7750	0.04	8	0.27	Small
HJHS Gr. B	T0–T1	−0.4888	0.62	7	0.18	Small
	T0–T2	−2.1082	0.03	7	0.80	Large
FISH Gr. A	T0–T1	2.3281	0.01	8	0.82	Large
	T0–T2	2.0404	0.04	8	0.72	Large
FISH Gr. B	T0–T1	2.3026	0.02	7	0.87	Large
	T0–T2	2.1205	0.03	7	0.80	Large
NRS Gr. A	T0–T1	−0.9567	0.33	8	0.34	Medium
	T0–T2	−1.9874	0.04	8	0.70	Large
NRS Gr. B	T0–T1	−0.2222	0.82	7	0.08	Negligible
	T0–T2	−2.1630	0.03	7	0.82	Large

## Data Availability

The raw data supporting the conclusions of this article will be made available by the authors, without undue reservation.

## Abbreviations

The following abbreviations are used in this manuscript:

HA	Haemophilic arthropathy
QoL	Quality of life
HJHS	Haemophilia Joint Health Score
FISH	Functional Independence Score in Haemophilia
NRS	Numerical Rating Scale
